# Head-Mounted Augmented Reality in the Planning of Cerebral Cavernous Malformation Resections: Demonstrating Feasibility Through an Initial Case Series

**DOI:** 10.3390/brainsci16101094

**Published:** 2026-10-10

**Authors:** Luke L. Jouppi, Clifford A. Pierre, Matias Luis Costa, Radwan I. Takroni, Nicholas Caffes, Stepan Capek, Aaron J. Gustin, Majid Aljoghaiman, Zane A. Tymchak, Akshal S. Patel, Stephen J. Monteith

**Affiliations:** 1 Seattle Science Foundation, Seattle, WA 98122, USA; 2 Spectrum Research Consulting, Tacoma, WA 98405, USA; 3 Department of Neurosurgery, Swedish Neuroscience Institute, Seattle, WA 98122, USA; 4 Department of Neurosurgery, The University of Texas Medical Branch at Galveston, Galveston, TX 77555, USA; 5 Department of Neurosurgery, Aseer Central Hospital, Abha 62523, Saudi Arabia; 6 Wellspan Neurosurgery, Wellspan York Hospital, York, PA 17403, USA; 7 Department of Neurosciences, Charleston Area Medical Center, Charleston, WV 25301, USA; 8 St Vincent Regional Hospital Brain and Spine, Billings, MT 59101, USA; 9 Surgery Department, College of Medicine, King Faisal University, Al-Hofuf 36362, Saudi Arabia

**Keywords:** augmented reality, cavernoma, cavernous malformation, case series, head-mounted augmented reality, microsurgery

## Abstract

**Highlights:**

**What are the main findings?**

- HMAR led to actionable changes in 3 (21.4%) of 14 CCM resection plans.
- Utilization of HMAR added a mean of 2.7 ± 1.0 min in the operating room.

**What are the implications of the main findings?**

- Trainees appraised HMAR as helpful in appreciating lesions’ vascular associations.
- HMAR enabled 3D visualization and richer spatial detail than conventional 2D MRI.

**Abstract:**

Objectives: Lesion localization, associated vascular structures, and corticotomy selection pose key challenges when planning cerebral cavernous malformation (CCM) resections in eloquent areas. Head-mounted augmented reality (HMAR) may improve visualization without the gaze disruptions imposed by conventional neuronavigation. This study investigated the preoperative feasibility of HMAR in surgical plan modifications and educational value. Methods: We performed a single-center retrospective review of all CCM patients with HMAR-assisted preoperative planning from 1 September, 2022, to 31 August, 2024. Three-dimensional reconstructions of patients’ magnetic resonance imaging were viewed preoperatively using HMAR. Registration accuracy was confirmed against conventional neuronavigation. Surgical plan modifications entailed alterations of the operative goal, trajectory, or steps. Educational value of HMAR was appraised by trainees as “not helpful” when only confirming existing plans, “helpful” when facilitating a greater understanding of the lesion, and “very helpful” when spurring additional positioning or plan modifications. Results: Of 19 patients with CCM resections, 14 patients with HMAR-assisted preoperative planning were included. The average age was 38.6 ± 14.5 years, with 6 males and 8 females. Three of 14 (21%) surgical approaches were modified following HMAR use: two to minimize cortical disruption and one to avoid injuring an associated developmental venous anomaly. Registration was accurate in all cases. Between registration and utilization, HMAR added an average of 2.7 ± 1.0 min in the operating room. The mean appraisal of the educational value of HMAR was “helpful.” Conclusions: Preoperative planning with HMAR prompted surgical plan modifications and often improved trainees’ understanding without introducing significant workflow disruptions. Three-dimensional visualization offered dynamic spatial details beyond traditional two-dimensional multiplanar MRI.

## 1. Introduction

The total resection of cerebral cavernous malformations (CCMs) in eloquent brain areas necessitates great precision in both preoperative planning and in technical execution. While complete resections are associated with lower rates of recurrent hemorrhage and symptoms, these operations require an in-depth understanding of the lesion’s tridimensional architecture and its spatial relationships to surrounding neuroanatomic structures [1]. The development of neuronavigational systems has offered great promise to facilitate idealized and accurate trajectories that minimize tissue disruption, though these systems impose a necessity for surgeons to periodically redirect their gaze from the operative field [2,3]. In the last two decades, head-mounted augmented reality (HMAR) has emerged as an adjunct or alternative to conventional neuronavigation that may obviate the need for such ergonomic disruptions [4,5,6]. Though HMAR systems have grown increasingly popular in medical education and appear promising as a preoperative planning tool, they remain understudied in the context of CCM resection [7,8]. This study aimed to investigate the feasibility of HMAR in the planning of CCM resections, to evaluate its impact on preoperative planning, and to determine its value in the education of trainees.

## 2. Materials and Methods

The authors performed a retrospective review of all patients who underwent craniotomies for CCM resection between 1 September 2022, and 31 August 2024. Head-mounted augmented reality was used as a surgical planning adjunct for all cases except emergencies and cases performed while the HMAR system was already in use. Only resections that utilized HMAR during preoperative planning were eligible for inclusion in the present study. Given the retrospective design, the requirements for informed consent to proceed were waived. This case series was reported in accordance with the PROCESS and CARE Guidelines [9].

Each patient underwent preoperative magnetic resonance imaging (MRI) of the brain with and without contrast as well as a head computed tomography (CT) scan without contrast. The resulting images were transferred from our institution’s Picture Archiving and Communication System to the Medivis Surgical AR Workstation (Medivis, New York, NY, USA). After connecting the HoloLens 2 AR headset to the Medivis AR station to allow direct three-dimensional visualization, the images were opened and processed as necessary (Microsoft, Redmond, WA, USA). After achieving satisfactory imaging results, the virtual hologram was saved on the workstation for further consultation in the operating room.

**Figure 1:**
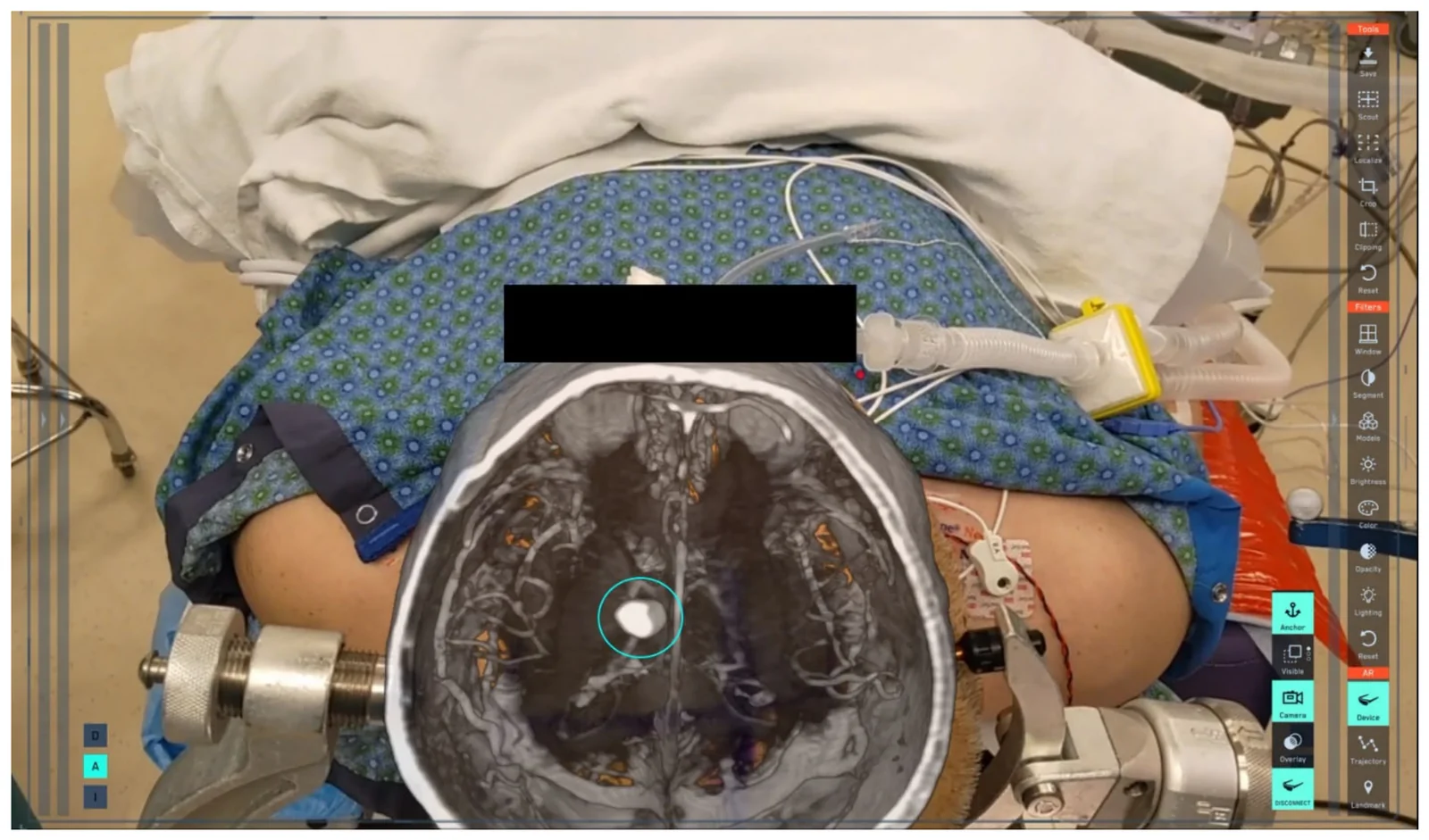
Example of a HMAR hologram demonstrating a CCM and allowing enhanced visualization of the lesion’s relationship to surrounding vasculature. The cyan circle highlights the targeted CCM and was added in post-processing to aid reader identification.

Once in the operating room, the virtual hologram was re-opened through the workstation and registered to the patient (Figure 1).

**Figure 2:**
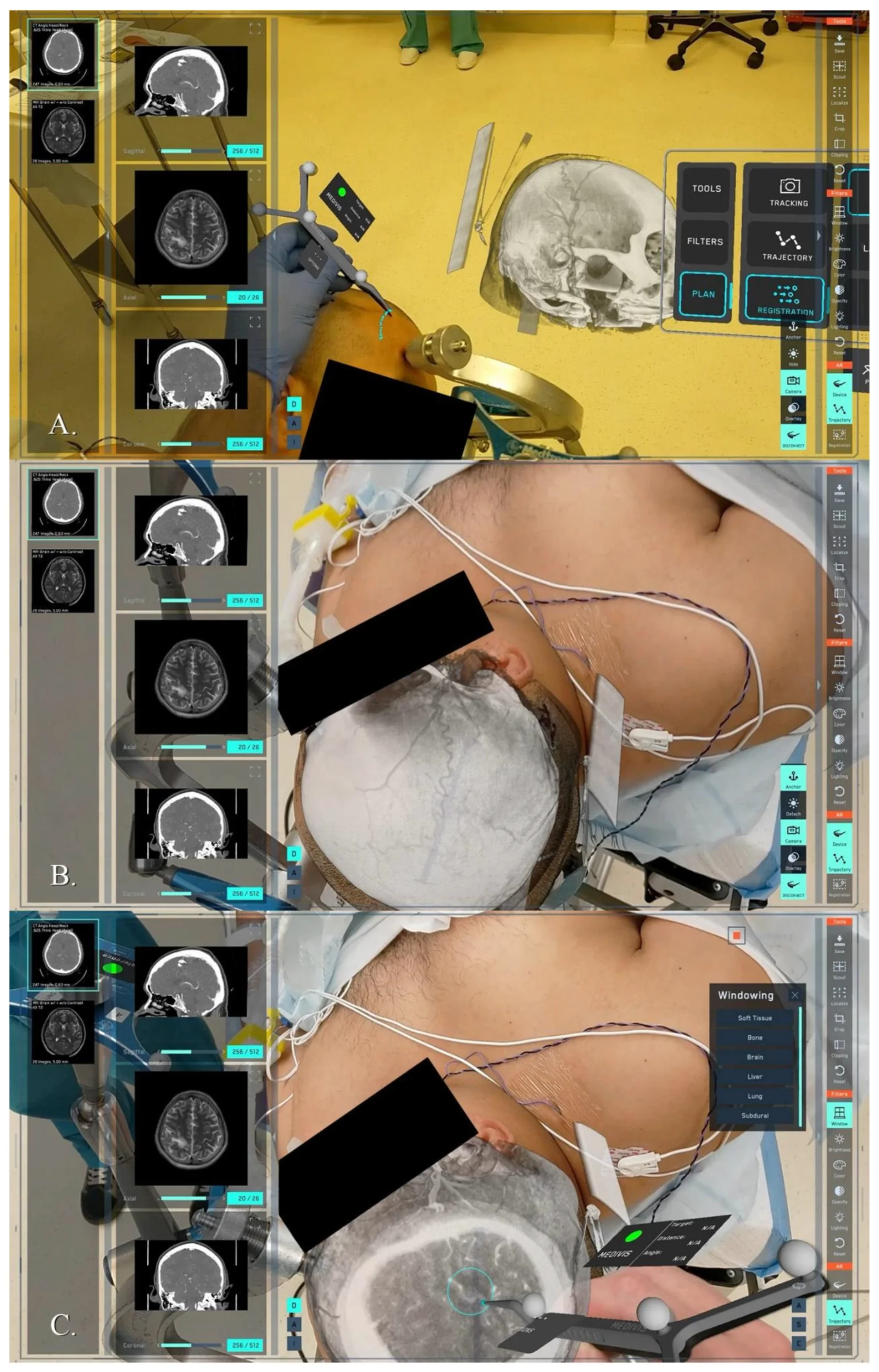
Registration of virtual 3D hologram to the patient’s anatomy using free-hand technique (**A**). Successful registration (**B**) allowed for preoperative visualization of the patient’s pathology using HMAR (**C**). The cyan outline in panel (**C**) was added to aid readers in identifying the site of the lesion.

Two methods were available to adjust the hologram to match the patient: free-hand registration and optical localization. The free-hand registration method involved the placement of physical landmarks on the patient’s surface anatomy for the primary configuration, with further adjustments made using hand gestures recognized by the HoloLens 2 to adjust hologram position and size as needed (Figure 2). Registration accuracy was confirmed by qualitative evaluation of the hologram’s conformity to key bony landmarks such as the nasion, lateral canthi, asterion, pterion, and inion.

The optical localization method involved a special tool recognized by the HoloLens 2 to match physical landmarks to virtual fiducials on the hologram. Calibration methodology was chosen on a case-by-case basis. In each case, registration and verification was performed by a single neurosurgeon and visualized by all members of the surgical team.

A standard skin incision was planned using standard Stealth neuronavigation (Medtronic Inc., Louisville, CO, USA). Following positioning of the patient and development of the surgical plan via conventional methods, the hologram was registered to the patient. This three-dimensional (3D) reconstruction was subsequently evaluated using the HMAR system; other screens in the operating room allowed visualization by all surgical team members. Positioning adjustments to the patient’s neck, bed height, and incision plan were made as necessary and verified with Stealth navigation. Following these adjustments, the surgical team discussed pertinent findings and whether any alterations to the surgical plan would be beneficial.

To determine the objective value of preoperative HMAR implementation, the surgical team noted whether modifications were made to the conventional plan after use of HMAR. Modifications were defined as alterations to the surgical goal, approach, or other aspects of the surgical plan. Registration accuracy was defined as the correlation of anatomical landmarks after registration between standard Stealth registration and HMAR. To assess clinical outcomes, modified Rankin Scales (mRS) were collected preoperatively and one month after the operation. Additional time spent in the operating room as a result of HMAR utilization was documented from operative recordings.

Our study defined educational value as the system’s capacity to facilitate deeper understanding of the patients normal and pathological anatomy, as well as pertinent surgical considerations of each case. Educational value was stratified into three categories: “not helpful,” “helpful,” and “very helpful.” Ratings of “not helpful” were applied in straightforward resections in which HMAR served only to confirm the planned approach. “Helpful” ratings were applied when HMAR facilitated better understanding of the lesion’s location, depth, or associated structures, but spurred no major alterations to the operative plan. “Very helpful” ratings denoted cases in which HMAR generated concrete changes in positioning, approach, or additional operative steps. For each case, independent assessments of educational utility were performed by a cerebrovascular fellow and an attending neurosurgeon, followed by discussion on the perceived utility of HMAR and inter-rater agreement.

## 3. Results

Between 1 September, 2022, and 31 August, 2024, 19 patients underwent open resection for CCM. Preoperative plans were assisted by HMAR for fourteen (73.7%) patients, rendering them eligible for inclusion. The mean age was 38.64 ± 14.52 years, with 6 males and 8 females. Standalone free-hand hologram image registration was used in 12 cases, while 2 cases employed virtual fiducial-based optical localization. In all cases, registration via HMAR was deemed accurate, matching the same anatomical landmarks as standard Stealth neuronavigation. The application of HMAR into preoperative planning led to changes in the surgical approach for 3 (21.4%) of the 14 CCM resections. Table 1 summarizes the outcomes, demographic information, and HMAR parameters for all cases.

Clinical outcomes were favorable, with the mean mRS improving from 1.71 ± 0.82 preoperatively to 0.79 ± 0.70 one month after the operation, though this data cannot be attributed to HMAR given the lack of comparison cohort. Total utilization of HMAR in the operating room, including both registration and visualization, added a mean of 2.73 ± 0.99 min. Augmented reality also was appraised as “helpful” (2.0 ± 0.78) in its average educational value, improving trainees’ understanding of the lesion’s position and relation to nearby structures. Supplemental HMAR use during preoperative planning was noted to be “very helpful” in 4 cases (28.6%) where it both improved trainees’ understanding and contributing to additional changes in positioning or operative characteristics. In the following, we present three illustrative cases to detail our experiences: two cases in which HMAR-guided planning led to surgical strategy modifications and one case where no modifications were made.

### 3.1. Case 1: Avoiding the Motor Cortex

**Figure 3:**
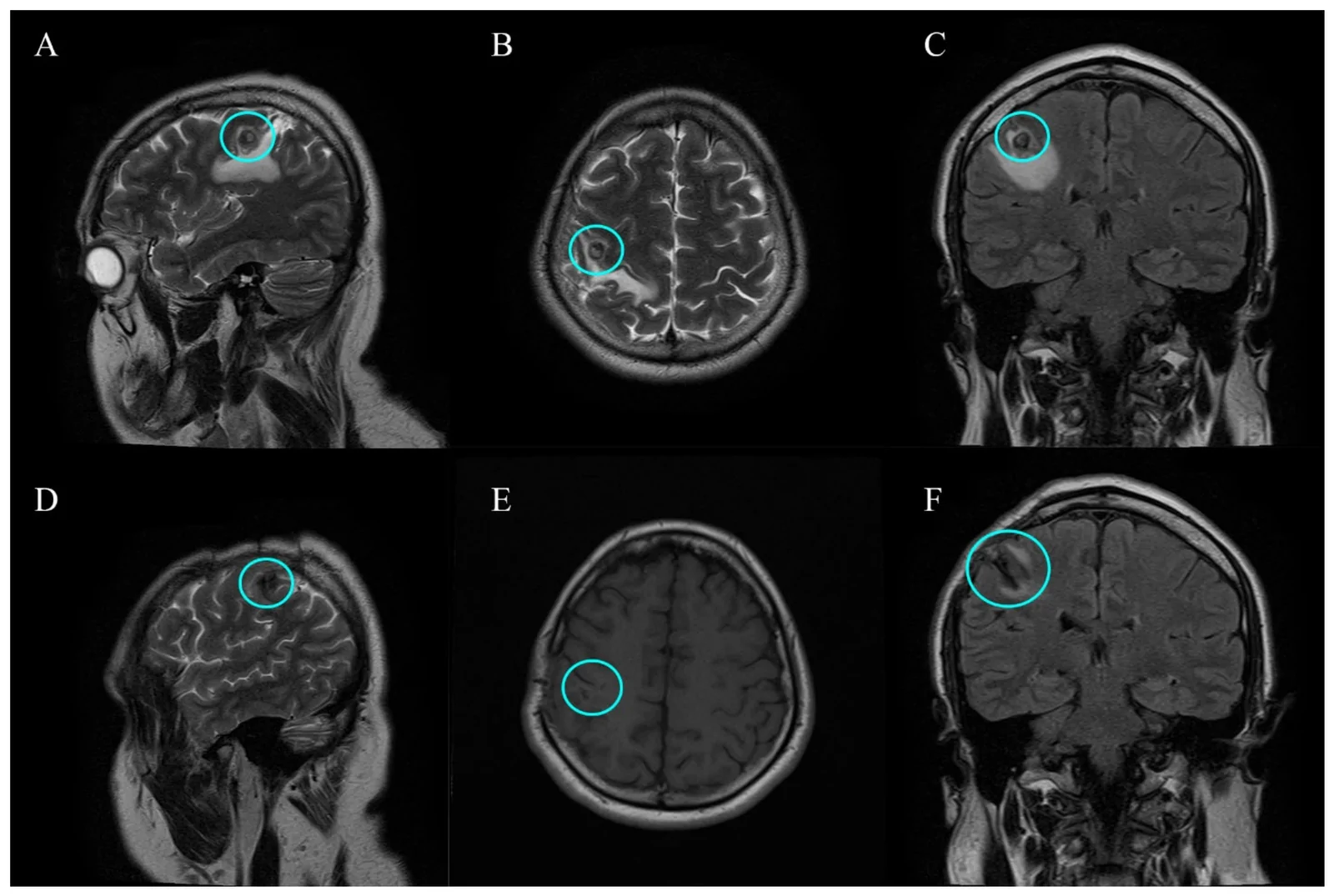
Preoperative (**A**–**C**) and postoperative (**D**–**F**) magnetic resonance imaging for the patient discussed in Case 1, in which a CCM developed directly under the motor cortex, as circled in blue. Preoperative utilization of HMAR resulted in a modification of the planned trans-sulcal approach to avoid vascular structures within the sulcus.

A patient in his late thirties with a previous history of AVM resection and stereotactic radiosurgery presented with frequent headaches and seizures that particularly affected the left arm despite antiseizure medications. Magnetic resonance imaging revealed a 7 × 6 × 10 mm CCM directly under the motor cortex with adjacent edema and hemorrhage (Figure 3).

Following a discussion of treatment options, a decision was made to proceed with surgical resection. During preoperative planning, 3D visualization using HMAR provided enhanced clarity of the lesion’s depth, location, and vascular relationships, providing a streamlined complement to conventional two-dimensional (2D) multiplanar MRI. Following this improved appreciation, the trans-sulcal approach was modified to optimize the avoidance of vascular structures in the sulcus and to utilize the most direct route to the CCM that would not compromise either the neighboring vasculature or the healthy adjacent motor strip. Complete en bloc resection was achieved with no complications. One year after the surgery, he reported improvements in the deficits of his left-handed dexterity and he was advised to continue occupational therapy in pursuit of complete symptom resolution. Otherwise, the patient demonstrated a good recovery, with his mRS decreasing from 2 preoperatively to 1 postoperatively.

### 3.2. Case 2: Avoiding a Developmental Venous Anomaly and the Vein of Labbe

**Figure 4:**
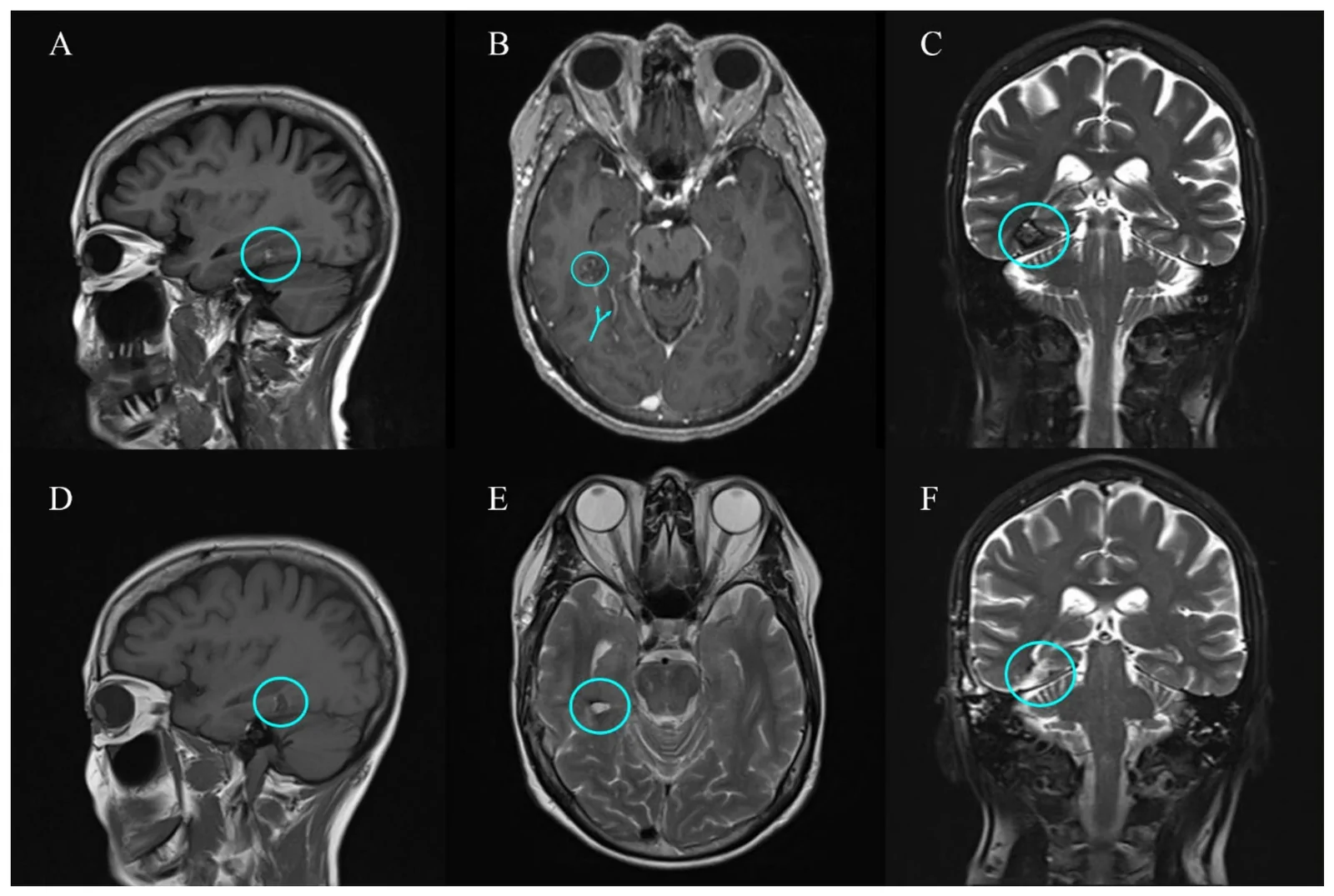
Preoperative (**A**–**C**) and postoperative (**D**–**F**) magnetic resonance imaging for Case 2. Preoperative application of HMAR better revealed the developmental venous anomaly indicated by the arrow in panel (**B**) that was associated with the lesion, thereby facilitating modification of the surgical approach to avoid its injury.

A patient in her late sixties who presented to the clinic with an incidentally found right temporal CCM (Figure 4).

She had a history of traumatic brain injury, headaches, ocular migraine, and transient visual deficits, but no personal or family history of either cavernoma or other vascular malformations. Repeat MRI demonstrated a microbleed which had occurred near the optic radiation, explaining her transient visual deficits. Following a discussion, she elected to proceed with surgical intervention. Application of HMAR during the planning of the operation resulted in clear visualization of the vein of Labbé and a developmental venous anomaly (DVA), which spurred minor alterations to the surgical plan. The surgical approach was modified to avoid injury to the DVA or stretching the vein of Labbé. Gross total resection was achieved with no complications. One year after surgery, the patient endorsed a good recovery and denied any ongoing visual deficits. In this case, the primary benefit of HMAR was the enhanced clarity 3D visualization provided regarding the lesion’s surrounding vasculature, informing an optimal approach.

### 3.3. Case 3: Trans-Sulcal Approach to a Right Occipitoparietal Cavernous Malformation

**Figure 5:**
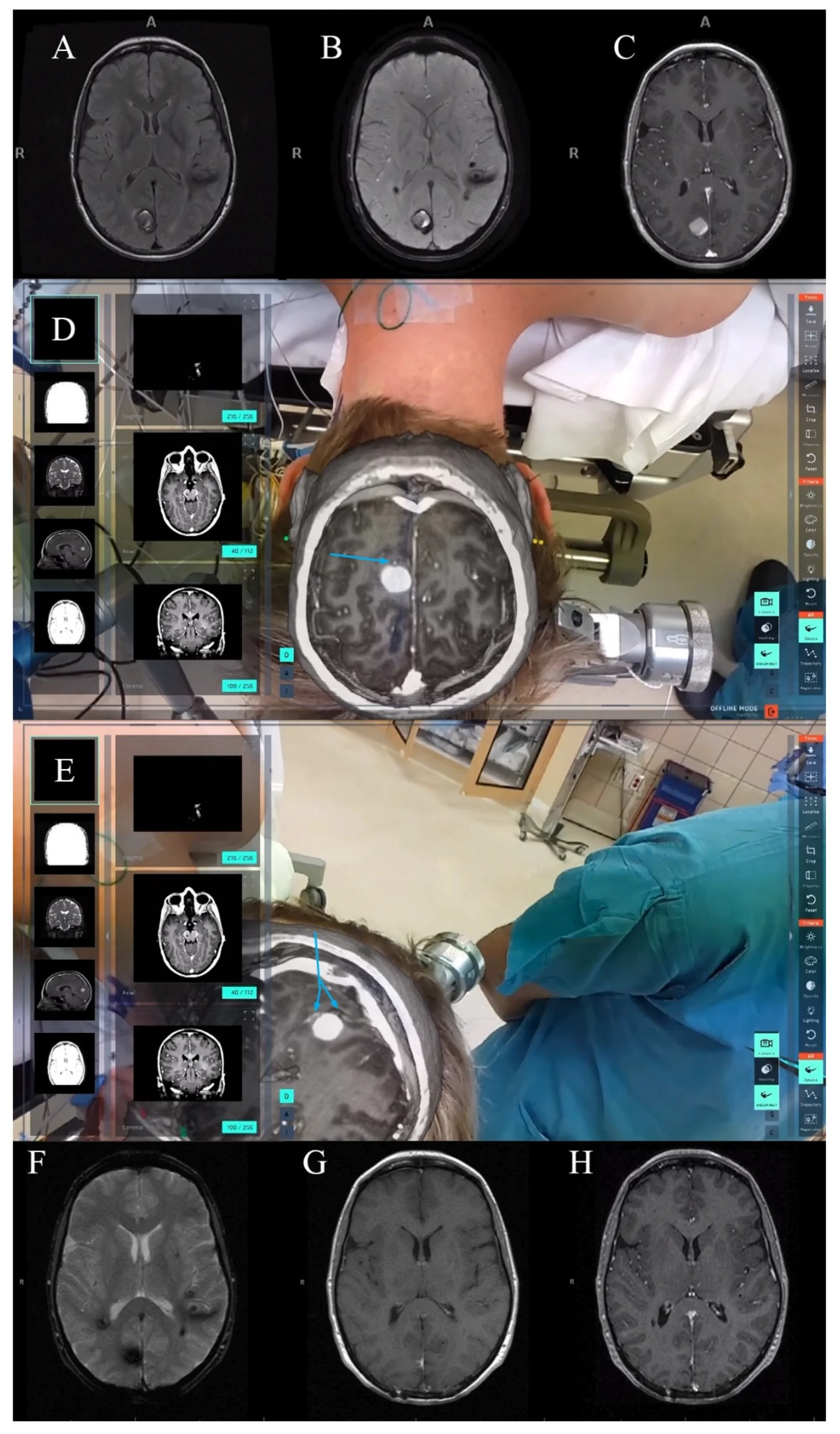
Preoperative (**A**–**C**) and postoperative (**F**–**H**) MRI for Case 3. Preoperative application of HMAR allowed for better visualization of the adjacent veins to the CCM indicated by arrows in panels (**D**,**E**). Complete resection of the lesion was obtained with no residual malformation.

The final case to be illustrated was a 36-year-old woman who had numerous CCMs incidentally discovered during workup for a transient episode of hearing loss. In addition to the natural history and course of CCMs, the use of propranolol was discussed given its reported potential to decrease the risk of hemorrhage in patients with cavernous malformations [10]. As she was asymptomatic, she declined medication and was followed with repeated MRIs for three years. At that point, serial imaging demonstrated interval bleeding of her right parietooccipital cavernoma. Coupled with the growth of this CCM from 1.7 to 2.1 cm, a decision was made to proceed with right parietooccipital craniotomy and resection due to concern for serious hemorrhage. During preoperative planning, HMAR provided enhanced visualization of nearby veins and a better appreciation of the surgical approach (Figure 5).

The operation was performed under microscopy with clear discoloration of the brain tissue. No transcortical route was used; instead, a trans-sulcal corridor was utilized. The CCM was identified, circumferentially dissected with bipolar cautery, and resected piecemeal along with the drainage of a large amount of old, chronic blood products. Gross total resection was obtained without complications. Two years following her operation, there were no new hemorrhages from any of the other CCMs. There was residual hemosiderin staining of the brain tissue outside of the resected cavernoma, though there was no residual or recurrence of the lesion itself. She remained asymptomatic after the procedure and endorsed a good recovery.

## 4. Discussion

The successful resection of CCMs in eloquent areas is contingent upon meticulous surgical planning and precise execution [1,10]. Historically, the introduction of neuronavigation at the turn of the century was perceived to improve visualization, aid in the selection of optimal trajectories, and enhance operator comfort, particularly for the resection of deeper lesions [2,3,11]. Conventional neuronavigation systems necessitate for the surgeon to alternate their gaze between the navigational display and the operative field [2,4]. Recently, HMAR has drawn increasing interest for its potential to provide similar improvements in visualization while allowing the surgeon to maintain their focus on the operative field [6,12,13]. In our series, preoperative analysis of the 3D hologram during preoperative planning and in the operating room allowed for rapid inspection of the lesion from any angle of interest, offering more degrees of freedom than the standard orthogonal planes used in multiplanar MRI with neuronavigation. While in the majority of cases (11/14, 78.5%) this alternative perspective only confirmed the pre-existing surgical plans derived from conventional approaches, HMAR did produce actionable modifications to the surgical plan in 3 of 14 (21.4%) cases, largely due to instances in which 3D HMAR was able to confer an enhanced appreciation of lesions’ surrounding vascular structures as compared to multiplanar MRI.

Head-mounted augmented reality is subject to several challenges similar to conventional neuronavigation, particularly in the time burden inherent to its preparation and the inability to account for intraoperative tissue deformation. However, when including both the time for registration and utilization, preoperative HMAR utilization added only a minimal average increase of 2.7 min in the operating room to cases in our series. Outside of the operating room, HMAR was able to be integrated into existing pre-operative workflows. Total utilization time averaged approximately 30 min per case, with minor variations dependent on the number of trainees present.

In the majority (10/14, 71.4%) of cases in our series, trainees appraised HMAR as either “helpful” or “very helpful,” even to cerebrovascular fellows with advanced neurosurgical training. In the four straightforward cases (28.6%) where HMAR was deemed “not helpful,” the technology was still felt to have facilitated more precise, tailored craniotomies. The increasing adoption of neuro-endovascular procedures and stereotactic radiosurgery has contributed to a decline in microsurgical case volumes in recent years [14,15]. While trainees in our series considered HMAR “helpful,” further studies investigating the quantifiable impacts of HMAR on surgical education may benefit future training paradigms amidst this changing landscape [16,17,18].

This study is subject to limitations. The relative rarity of CCMs resulted in a constrained number of cases for analysis, rendering a case series as the most suitable study design and preventing more thorough statistical analysis. Registration was performed by a single neurosurgeon in each case; as registration accuracy was not able to be tracked via quantitative methods, accuracy was qualitatively evaluated by cerebrovascular fellows and an attending neurosurgeon using bony landmarks. While this study demonstrated the feasibility of HMAR in surgical planning, whether and to what extent HMAR influenced that improvement remains unclear; the lack of a comparator group precludes this study from establishing conclusive evidence for superior planning or patient outcomes between HMAR or conventional neuronavigation. Future research with a greater sample size is needed for conclusive determination of the impacts of HMAR-assisted surgical planning on patient outcomes. Furthermore, during the study period of this article, intraoperative utility of HMAR was not yet cleared by the United States Food and Drug Administration (FDA), so the system was required to be removed prior to the first incision. Now that this system has been approved by the FDA for intraoperative use in cranial applications, its use case as an adjunct to traditional neuronavigation systems is more compelling.

## 5. Conclusions

Head-mounted augmented reality for preoperative planning enhanced the visualization of CCMs and their associated structures, informed major alterations to the surgical plan in 3 of 14 (21%) cases, and often improved trainees’ educational experience. The primary benefits of HMAR were found in enabling 3D visualization, which provided richer spatial detail than conventional 2D views in multiplanar MRI. On average, HMAR utilization added less than three minutes to surgeon workflows in the operating room. Our series highlights the feasibility of the adoption of HMAR for preoperative planning, though benefits regarding clinical outcomes cannot be attributed to HMAR given the lack of comparison cohort in this study. Future studies are needed to quantify clinical benefits and to determine intraoperative utility.

## Figures and Tables

**Table 1:** Patient Characteristics and Trainee Appraisal of HMAR.

Case Number	Age	Sex	Additional Time in the Operating Room Related to HMAR (min)	Lesion Location	Reported Symptoms	Bleeding	PreOP MRS	PostOP mRS (1 Month)	Surgical Plan Modifications	Benefits & Limitations	Educational Value (1–3)
1	31	F	3.9	Right superior temporal gyrus	Seizures	Yes	1	1	No	HMAR allowed for a small, tailored craniotomy.	Not helpful
2	34	M		Left temporal lobe	Headache	Yes	2	1	No	HMAR helped appreciate the depth of the lesion in relation to the cortex, which made it more predictable to find during surgery.	Helpful
3	30	F		Cerebellum	Right hand paresthesias and headache	Yes	2	1	No	Augmented reality helped to confirm the plan for the trajectory of the left suboccipital approach.	Not helpful
4	22	F	2	Right temporoparietal	Headaches, nausea, and auditory disturbances	Yes	1	0	No	Helped to tailor a small craniotomy and confirm the approach.	Not helpful
5	69	F	3.3	Right temporal	Visual changes, headaches, and unsteadiness	Yes	2	1	Yes	HMAR allowed the clear visualization of the vein of Labbé and DVA; it led to modulation of the angle of approach to avoid imparting any stretch on the vein of Labbé.	Very helpful
6	35	M		Right temporal	Seizures	No	1	0	No	HMAR helped make the craniotomy safer. It helped us appreciate the depth of the lesion in relation to the cortex and increased our awareness of the location of a large cortical vein we had expected.	Helpful
7	38	M	2.1	Right parietal	Headaches, weakness of the left arm, intermittent focal motor seizures	Yes	2	1	Yes	HMAR resulted in a plan modification. We used a trans-sulcal approach to the cavernous malformation in order to minimize disruption of the motor cortex directly above the lesion, and HMAR helped us more optimally avoid vascular structures within the sulcus. It also helped us plan for the most direct route to the lesion to avoid injury to the motor strip.	Very helpful
8	46	F	4.2	Pontine	Vertigo, difficulty with balance, diplopia	Yes	3	2	No	HMAR helped visualize the working corridor and trajectory to middle cerebellar peduncle and to the brainstem cavernoma.	Very helpful
9	32	M		Left frontal	Seizures and loss of consciousness	No	3	1	Yes	HMAR helped to identify the appropriate cortical trajectory to the lesion.	Helpful
10	20	M		Right occipital	Vision loss and seizures	Yes	2	0	No	HMAR helped confirm the craniotomy planning, area of corticotomy, and DVA location.	Helpful
11	37	F		Left cerebellar	Fatigue, headaches, memory difficulties, left-sided ataxia, and visual changes	Yes	2	2	No	HMAR helped provide a better understanding of the perforating vessels from the nearby PICA. It also helped us orient the angle of the microscope in relation to the patient’s shoulder to reach the target lesion.	Helpful
12	69	F	3.2	Left cingulate gyrus	Sudden and severe headaches	Yes	2	0	No	HMAR helped explore the anatomy in 3D prior to the case, which in turn, helped to better prepare us for the case. It was helpful for the planning of positioning, incision, craniotomy, and trajectory.	Very helpful
13	38	M		Left frontal	Seizure, Speech difficulty	Yes	1	1	No	This case was a relatively simple craniotomy for resection of a left frontal cavernous malformation. The implementation of HMAR helped to tailor the craniotomy, but did not provide much value beyond this.	Not helpful
14	40	F		Right occipitoparietal	None	Yes	0	0	No	We did not make alterations to the surgical plan for a trans-sulcal approach due to HMAR use, though it did help us appreciate the proximity and relationship to nearby veins.	Helpful

## Data Availability

The data supporting this study’s findings are available from the corresponding author upon reasonable request, subject to institutional data-sharing policies.

## Abbreviations

The following abbreviations are used in this manuscript:

CCM	Cerebral Cavernous Malformation
HMAR	Head-mounted augmented reality
MRI	Magnetic Resonance Imaging
CT	Computed Tomography
3D	Three-dimensional
2D	Two-dimensional
mRS	Modified Rankin Scale
DVA	Developmental venous anomaly
FDA	United States Food and Drug Administration
